# 108. Immune Status of Indian Pediatric Health Care Workers against Various Vaccine Preventable Diseases

**DOI:** 10.1093/ofid/ofac492.186

**Published:** 2022-12-15

**Authors:** Sanjay Verma, Ravinder Kaur Sachdeva, Vidushi Mahajan, Amit Rawat, Urvashi Nehra, Vignesh Subramani, Monika Jassal, Bhavneet Bharti

**Affiliations:** PGIMER, Chandigarh, India, Chandigarh, Chandigarh, India; PGIMER, Chandigarh, India, Chandigarh, Chandigarh, India; GMCH, CHANDIGARH, Chandigarh, Chandigarh, India; PGIMER, CHANDIGARH, INDIA, Chandigarh, Chandigarh, India; PGIMER, CHANDIGARH, INDIA, Chandigarh, Chandigarh, India; PGIMER, CHANDIGARH, INDIA, Chandigarh, Chandigarh, India; GMCH, CHANDIGARH, Chandigarh, Chandigarh, India; PGIMER, Chandigarh, India, Chandigarh, Chandigarh, India

## Abstract

**Background:**

Health care workers (HCWs) possess a potential risk to acquire and spread various infections. This study was planned to assess the immune status of HCWs in pediatric departments of two tertiary care hospitals in Northern India.

**Methods:**

In this cross-sectional study, HCW’s (Indians), working in pediatric departments of these hospitals, over 6-months period (July18-Dec18) were enrolled after taking written consent and their 5-ml venous blood sample was collected. Ethical clearance was obtained from Institute Ethics committee, before enrolling subjects. Serum were tested for antibodies against diphtheria-toxin (DT), pertussis-toxin (PT), measles, mumps, rubella, varicella, hepatitis-B (HbsAb) and hepatitis-A using commercial IgG (quantitative) ELISA kits.

**Results:**

A total of 160 HCW’s (M:F=77:83), having mean age 30.6±7.8 years, were enrolled. Out of them 106 (66.3%) were resident doctors, 31 (19.4%) nursing-staff, 18 (11.3%) faculty members, 3 (1.9%) research-staff and 2 (1.3%) paramedical-staff. In our study, antibodies (IgG) against DT were between 0.01 to 0.1 IU/ml in 78.1% (120/160); requiring a Tdap booster; while 7 out of 125 had titers < 0.01 IU/ml, which needed 3-doses of primary vaccination. Antibodies (IgG) against PT < 30 IU/ml were seen in 60.6% (97/160), which were considered as seronegative, as per kit recommendations.

A total of 3% (5/160), 13.1% (21/160), 10% (16/160), 17.5% (28/160) had titers < 12 IU/ml for IgG antibodies against measles, mumps, rubella and varicella respectively; were considered unprotected. A total of 25% (40/160) had Anti-HBs antibody titers < 20 mIU/ml; which were low, therefore were advised to take one booster dose of Hep-B vaccine. A total of 15.6% (25/160) had IgG antibodies against hepatitis-A < 10 mIU/ml; were unprotected.

**Conclusion:**

Alarming proportions of pediatric-HCWs had low antibody titres against DT (78.1%) and PT (60.6%), necessitating a dose of Tdap. A total of 10%, 17.5% and 15.6% lacked protective antibodies against rubella, varicella and hepatitis-A. A quarter of screened population had low anti-Hbs titres, requiring boosting of immunity. Our study emphasizes the urgent need for improving immunization status of pediatric HCW’s; as they continue to remain susceptible to various vaccine preventable infectious diseases.

**Disclosures:**

**All Authors**: No reported disclosures.

